# Radiomics Nomogram Based on Multiple-Sequence Magnetic Resonance Imaging Predicts Long-Term Survival in Patients Diagnosed With Nasopharyngeal Carcinoma

**DOI:** 10.3389/fonc.2022.852348

**Published:** 2022-04-07

**Authors:** Kai Liu, Qingtao Qiu, Yonghui Qin, Ting Chen, Diangang Zhang, Li Huang, Yong Yin, Ruozheng Wang

**Affiliations:** ^1^Department of Head and Neck Comprehensive Radiotherapy, Affiliated Tumor Hospital of Xinjiang Medical University, Urumqi, China; ^2^Department of Radiation Oncology, Shandong Cancer Hospital and Institute, Shandong First Medical University and Shandong Academy of Medical Sciences, Jinan, China

**Keywords:** nasopharyngeal carcinoma, radiomics, overall survival, prediction model, nomogram, multiple-sequence MRI

## Abstract

**Purpose:**

Although the tumor–node–metastasis staging system is widely used for survival analysis of nasopharyngeal carcinoma (NPC), tumor heterogeneity limits its utility. In this study, we aimed to develop and validate a radiomics model, based on multiple-sequence magnetic resonance imaging (MRI), to estimate the probability of overall survival in patients diagnosed with NPC.

**Methods:**

Multiple-sequence MRIs, including T1-weighted, T1 contrast, and T2-weighted imaging, were collected from patients diagnosed with NPC. Radiomics features were extracted from the contoured gross tumor volume of three sequences from each patient using the least absolute shrinkage and selection operator with the Cox regression model. The optimal Rad score was determined using 12 of the 851 radiomics features derived from the multiple-sequence MRI and its discrimination power was compared in the training and validation cohorts. For better prediction performance, an optimal nomogram (radiomics nomogram-MS) that incorporated the optimal Rad score and clinical risk factors was developed, and a calibration curve and a decision curve were used to further evaluate the optimized discrimination power.

**Results:**

A total of 504 patients diagnosed with NPC were included in this study. The optimal Rad score was significantly correlated with overall survival in both the training [C-index: 0.731, 95% confidence interval (CI): 0.709–0.753] and validation cohorts (C-index: 0.807, 95% CI: 0.782–0.832). Compared with the nomogram developed with only single-sequence MRI, the radiomics nomogram-MS had a higher discrimination power in both the training (C-index: 0.827, 95% CI: 0.809–0.845) and validation cohorts (C-index: 0.836, 95% CI: 0.815–0.857). Analysis of the calibration and decision curves confirmed the effectiveness and utility of the optimal radiomics nomogram-MS.

**Conclusions:**

The radiomics nomogram model that incorporates multiple-sequence MRI and clinical factors may be a useful tool for the early assessment of the long-term prognosis of patients diagnosed with NPC.

## Introduction

Nasopharyngeal carcinoma (NPC) is a common malignant tumor among Chinese and Asian populations but rarely occurs in Europeans and Americans (1–3). The incidence of NPC varies significantly by region and ethnicity. China accounts for approximately 47.7% of all new NPC cases worldwide, which is ~20 times the global incidence (4–6). Comprehensive treatment based on concurrent radio-chemotherapy is the standard first-line treatment for locally advanced nasopharyngeal carcinoma; patients usually have long-term survival (7, 8). Although the local control rate is significantly improved (>90%), approximately 10%–15% of patients with advanced nasopharyngeal carcinoma develop local recurrence or distant metastasis, which reduces the overall survival (OS) rate (9, 10).

Survival risk assessment and treatment strategy decisions for NPC are mainly based on the tumor–node–metastasis (TNM) staging system (11). However, treatment outcomes vary widely among patients diagnosed at the same clinical stage (12), which suggests a need for a more effective method to determine the inherent biological heterogeneity within the tumor region. Radiomics refers to the high-throughput extraction and analysis of a large number of advanced and quantitative imaging features from medical images such as computed tomography (CT), positron emission tomography (PET), and magnetic resonance imaging (MRI), to quantify tumors (13, 14). Head and neck MRI is the main imaging modality routinely used for diagnosing and staging NPC (15, 16). However, in recent years, several studies have shown that MRI radiomics is a more accurate and reliable tool to evaluate treatment responses (17, 18) and for survival analyses (19, 20).

It is, therefore, reasonable to hypothesize that MRI radiomics can be used to predict the probability of OS. However, to the best of our knowledge, there are few MRI-based radiomics studies that investigate the NPC prognosis in areas with a low incidence of NPC such as Xinjiang, China. Therefore, this study aimed to develop and validate a radiomics model using multiple-sequence (MS)-MRI to estimate the OS probability for patients diagnosed with NPC.

## Materials and Methods

### Study Design and Workflow

**Figure 1:**
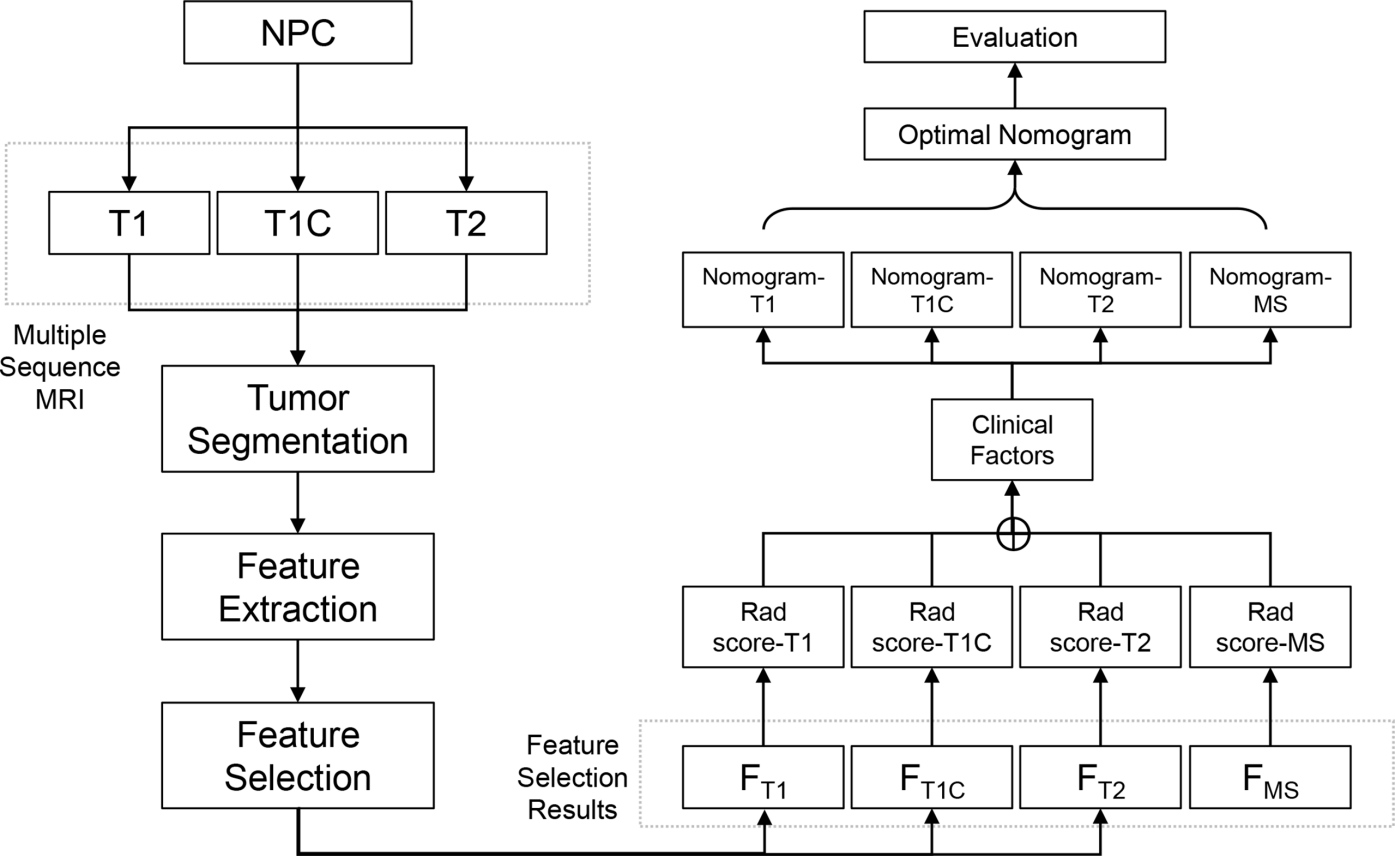
The study design and workflow.

The study design and workflow are illustrated in **Figure 1**. Patients who were diagnosed and treated for NPC were enrolled in the study and MS-MRIs were collected for radiomics analysis. The radiomics features were extracted and selected based on their clinical effectiveness in predicting survival. Rad scores and nomograms derived from each MRI sequence were established and compared, and the discrimination power of the optimized nomograms was evaluated.

### Patients

A total of 504 consecutive NPC patients who received treatment between March 2013 and June 2021 at the Affiliated Cancer Hospital of Xinjiang Medical University were enrolled in the study. Clinical risk factors associated with NPC, including age, sex, smoking, TNM stage, and clinical stage before treatment, were recorded for the enrolled patients. The inclusion and exclusion criteria are described in the **Supplementary Methods**. The study was conducted in accordance with the Declaration of Helsinki (as revised in 2013). Ethical approval was obtained from the Institutional Review Board of the Affiliated Cancer Hospital of Xinjiang Medical University (No. K-2021022).

### MS-MRI

Pretreatment MS-MRIs, including T1-weighted, T1 contrast (T1C), and T2-weighted sequences, were collected from all enrolled patients. MRI scans were acquired using a 3.0-T MRI scanner (MAGNETOM Verio, Siemens Healthineers, Germany). T1 images were acquired with the following protocols: repetition time (TR), 2,000 ms; echo time (TE), 9 ms; slice thickness, 5 mm; matrix size, 228 × 320; and in-plane resolution, 0.656 × 0.656 mm^2^. T1C images were acquired using the following protocols: TR, 2,000 ms; TE, 9 ms; slice thickness, 4 mm; matrix size, 301 × 320; in-plane resolution, 0.656 × 0.656 mm^2^; and scan after administration of the contrast agent, gadopentetate dimeglumine, 15 s. T2 images were acquired with the following protocols: TR, 4,000 ms; TE, 94 ms; slice thickness, 4 mm; matrix size, 320 × 320; and in-plane resolution, 0.656 × 0.656 mm^2^.

### Tumor Segmentation

**Figure 2:**
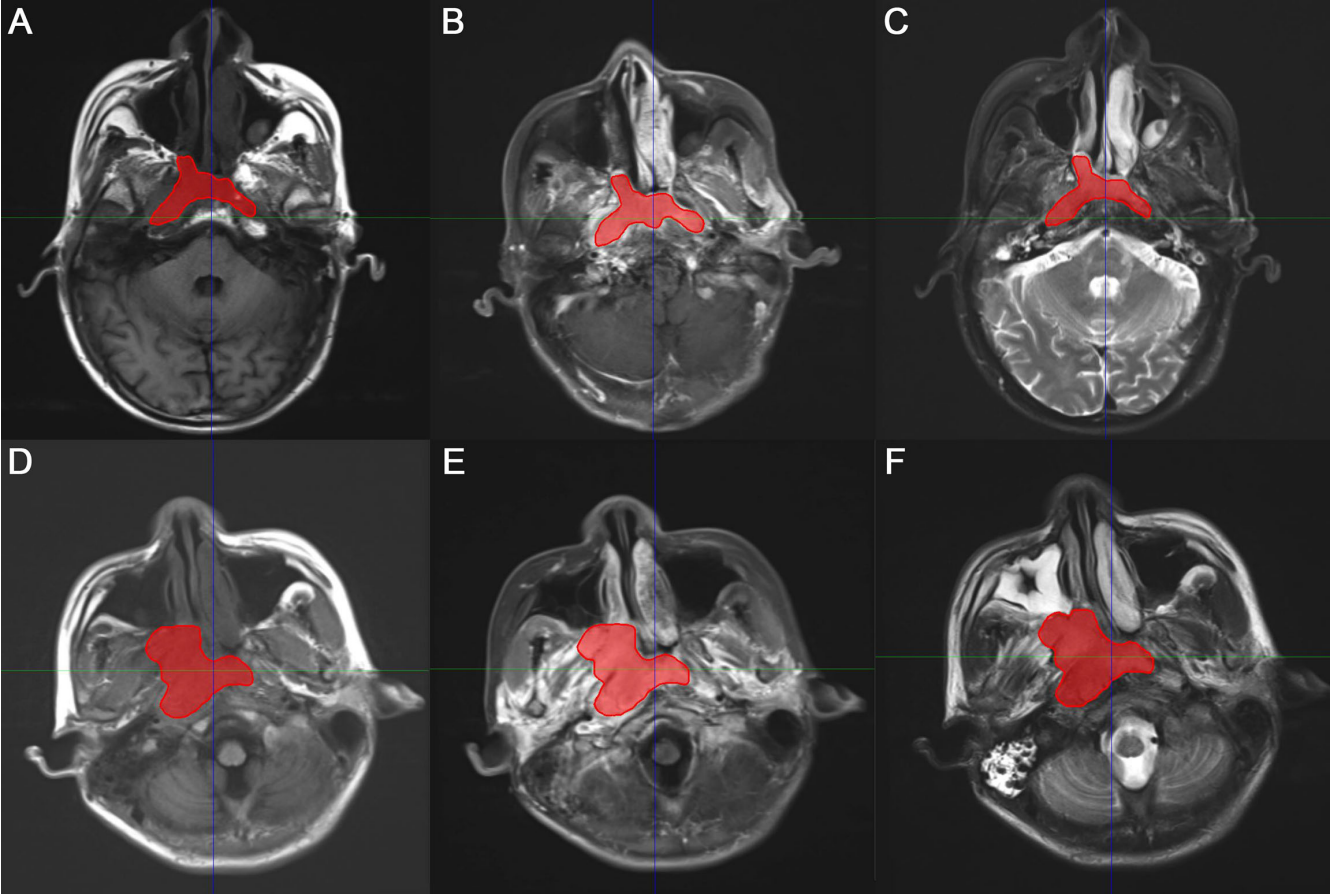
Representative two examples of contoured gross tumor volume (GTV) on multiple-sequence MRI. **(A, D)** T1-weighted, **(B, E)** T1 contrast, **(C, F)** T2-weighted MRI for patient 1 and 2, respectively.

The gross tumor volume (GTV), defined as the tumor region visualized on MRI images, was delineated by two experienced radiologists and oncologists on T1C sequence MRI using the AccuContour software (version 3.0, Manteia Medical Technologies Co. Ltd., Xiamen, China). The contoured GTVs were transferred into T1 and T2 sequences using rigid registration, and another experienced oncologist checked and modified the transferred results slice by slice. Examples of contoured GTV on MS-MRI are shown in **Figure 2**.

### Feature Extraction

Radiomics features were extracted using an embedded radiomics computational module-based PyRadiomics package that enables feature calculation in the AccuContour software (version 3.0, Manteia Medical Technologies Co. Ltd., Xiamen, China). A total of 851 radiomics features were extracted including i) 14 shape features, ii) 18 first-order intensity histogram-based and statistical matrix-based features divided into iii) 24 gray-level co-occurrence matrix (GLCM)-based features, iv) 16 gray-level run-length matrix-based features, v) 16 gray-level size zone matrix-based features, vi) 5 neighboring gray-tone difference matrices, and vii) 14 gray-level dependence matrix features. A total of 744 wavelet-based features (including intensity histogram and statistical matrix features) were also extracted from 8 wavelet decompositions.

### Radiomics Signature and Nomogram Construction

Before the radiomics signature was developed, feature selection was implemented to ensure that the signature was robust and effective. To identify the features that were most predictive of survival, the least absolute shrinkage and selection operator (LASSO) was performed using a Cox multiple variable regression model, a 10-fold cross-validation method, a “C-index” loss measurement, and non-normalized data.

After feature selection, the radiomics signature, also called the Rad score, was developed from a linear combination of selected features and corresponding coefficients derived from the LASSO. To determine whether the Rad score increased the ability to predict survival, the nomogram constructed by incorporating the Rad scores and clinical risk factors was compared with the nomograms constructed with clinical risk factors alone.

### Validation of the Radiomics Signature and Nomogram

The NPC patients were divided into a high-risk and a low-risk group according to the threshold or cutoff point of the Rad scores generated using the X-tile software (version 3.6.1). Patients with a Rad score above the cutoff point were placed in the high-risk group, and patients with a Rad score equal to or lower than the cutoff point were placed in the low-risk group. We then assessed the correlations between the Rad scores or nomograms and the OS in each risk group to determine their predictive power.

### Statistical Analysis

Statistical analyses were performed using the stats, rms, and *glmnet* packages of the R software (version 3.3.1). Mann–Whitney *U* tests or two-sample *t*-tests were used to compare the patients’ characteristics where appropriate. A univariate analysis was performed on the clinical risk factors used to generate the nomogram. For validation of the radiomics signature and nomograms, Kaplan–Meier survival analyses were used to evaluate the correlation between the Rad scores and OS. The log-rank test was used to measure differences in the survival curves between the low-risk and high-risk groups. Harrell’s concordance index (C-index) was used to evaluate the agreement between the predicted and actual OS probabilities of the Rad score and nomogram. A calibration curve was used to determine the optimal nomogram with the highest discrimination power, and the Hosmer–Lemeshow test was used to assess the agreement between the predicted and actual OS; *p* ≥0.05 was indicative of good agreement. The statistical significance level was set at *p <*0.05.

## Results

### Patient Characteristics

The 504 consecutive patients enrolled in this study were divided into training and validation cohorts, with 353 and 151 patients (7:3 ratio) in each cohort, respectively. The patient characteristics including clinical factors, treatment regimen, and follow-up information are summarized in **Table 1**. There were no significant differences between the cohorts for the variables assessed (*p* > 0.05).

**Table 1:** Demographic and clinical characteristics of patients with NPC in the training cohort and validation cohort.

Characteristic	Training cohort	Validation cohort
Gender		
Male	243 (68.8)	106 (70.2)
Female	110 (31.2)	45 (29.8)
Age (years)		
Mean	49.22	47.59
Range	9–85	10–80
Smoking		
Yes	126 (35.7)	55 (36.4)
No	227 (64.3)	96 (63.6)
T stage		
T1	20 (5.7)	6 (4.0)
T2	58 (16.4)	28 (18.5)
T3	172 (48.7)	76 (50.3)
T4	103 (29.2)	41 (27.2)
N stage		
N0	11 (3.1)	2 (1.3)
N1	38 (10.8)	16 (10.6)
N2	223 (63.2)	101 (66.9)
N3	81 (22.9)	32 (21.2)
M stage		
M0	326 (92.4)	136 (90.1)
M1	27 (7.6)	15 (9.9)
Clinical stage		
I	1	1
II	11	4
III	168	75
IVa	146	56
IVb	27	15
Pathological type		
NKDC	127	47
NKUC	224	103
Other	2	1
Induced chemotherapy		
Yes	310	136
No	43	15
Concurrent chemoradiotherapy		
Yes	323	141
No	30	10
Follow-up time (months)		
Median	34.8	38.4
Range	0.3–101.7	0.3–101.2

All data except age in the above table are numbers of patients, with percentages in parentheses. No difference was found between the training cohort and the validation cohort in either the clinical characteristics or recurrence status (p = 0.176–0.829).

NKDC, non-keratinizing differentiated carcinoma; NKUC, non-keratinizing undifferentiated carcinoma; Other, adenocarcinoma or small cell carcinoma.

### Radiomics Signature Construction and Validation

**Figure 3:**
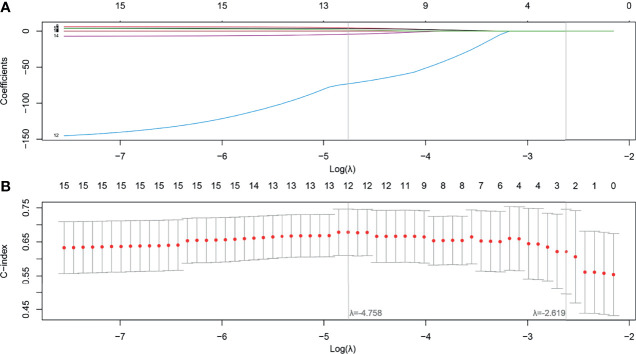
Feature selection using the least absolute shrinkage and selection operator (LASSO) with a Cox regression model. **(A)** Plot of the LASSO coefficient vs. log(λ). **(B)** Plot of the tuning parameter vs. log(λ). The C-indices are depicted with the corresponding λ. Vertical lines are maximum and 1 − standard criteria, respectively.

After feature selection using the LASSO Cox model, radiomics features 5, 5, and 7 were retained for T1, T1C, and T2 MRI images, respectively. When the selected features from each sequence were combined, 12 of 17 radiomics features were determined to be the most predictive radiomic features of the MS (**Figure 3**). Rad scores constructed from the T1, T1C, T2, and MS sequences were named Rad score-T1, Rad score-T1C, Rad score-T2, and Rad score-MS (the optimal Rad score), respectively. The formula for Rad score-MS is as follows:

**Table:** 

Rad score-MS =
T1.original.shape.Maximum2DDiameterRow * 0.016595576
+ T1.wavelet.LLH.glcm.SumAverage * 0.001760506
− T1.wavelet.LHL.glcm.JointAverage * 0.029007721
+ T1C.original.shape.MeshVolume * 2.12E-07
+ T1C.wavelet.LHL.firstorder.Median * 0.020523801
+ T1C.wavelet.HLL.glcm.InverseVariance * 4.652634819
+ T1C.wavelet.HLH.glszm.LargeAreaLowGrayLevelEmphasis * 7.97E-08
− T2.wavelet.LLH.ngtdm.Coarseness * 73.17990967
− T2.wavelet.LHL.glcm.InverseVariance * 4.408556368
+ T2.wavelet.LHL.glcm.MaximumProbability * 3.579222726
+ T2.wavelet.LHH.firstorder.Maximum * 0.00252154
+ T2.wavelet.HHL.glcm.MaximumProbability * 1.608499229

**Figure 4:**
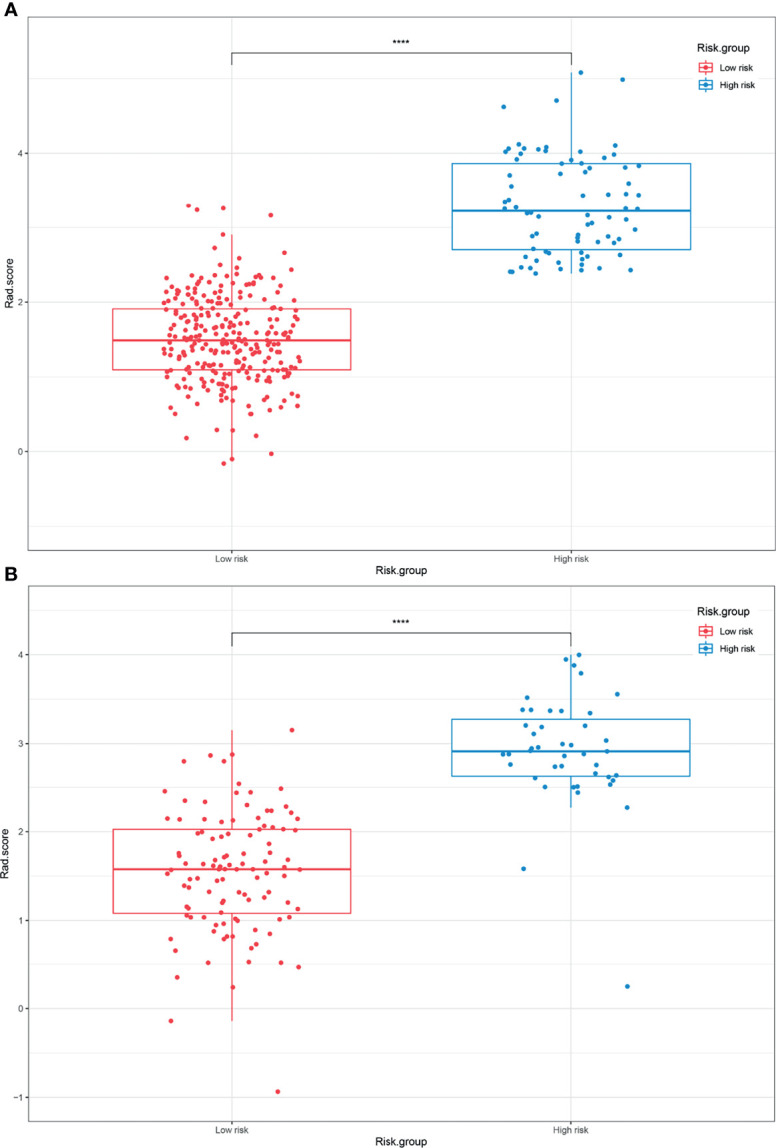
Box plots of the low- and high-risk groups subdivided based on the Rad score-MS in the **(A)** training cohort and **(B)** validation cohort. ****p < 0.0001.

**Figure 5:**
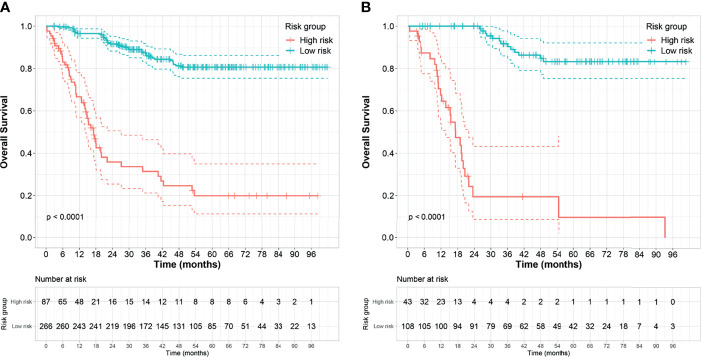
Kaplan–Meier survival analyses of the high- and low-risk groups subdivided based on the Rad score-MS in the **(A)** training cohort and **(B)** validation cohort. Dashed line indicates the two-sided confidence interval (CI) of the survival curves.

The optimal cutoff points generated from the X-tile software for Rad score-T1, Rad score-T1C, Rad score-T2, and Rad score-MS that were used to allocate the patients into low- and high-risk groups were 0.14, 0.38, 0.87, and 2.38, respectively. The Rad score-MS values for the two risk groups are shown in box plots in **Figure 4**. Significant differences between risk groups were observed for all Rad scores (all; *p* < 0.0001). The Kaplan–Meier curves depicted in **Figure 5** also revealed significant differences in the Rad score-MS between the low- and high-risk groups in both the training and validation cohorts (p < 0.0001). The formulas, box plots, and Kaplan–Meier results for Rad score-T1, Rad score-T1C, and Rad score-T2 are presented in the **Supplementary Results**. The C-indices for the established Rad scores demonstrate that Rad score-MS is superior compared with the other three Rad scores in predicting the risk level of OS for NPC patients in both the training and validation cohorts (**Table 2**).

**Table 2:** Comparison of the discriminating performance of the Rad score clinical nomogram and radiomics nomograms built by incorporating clinical risk factors and Rad score calculated from different MRI sequences.

Model	Training cohort		Validation cohort	
	C-index	95% CI	C-index	95% CI
Rad score-T1	0.626	0.602–0.650	0.599	0.562–0.636
Rad score-T1C	0.592	0.568–0.616	0.613	0.578–0.648
Rad score-T2	0.656	0.631–0.681	0.575	0.536–0.614
Rad score-MS	0.731	0.709–0.753	0.807	0.782–0.832
Clinical nomogram	0.766	0.744–0.788	0.743	0.724–0.762
Radiomics nomogram-T1	0.789	0.768–0.810	0.795	0.781–0.809
Radiomics nomogram-T1C	0.795	0.775–0.815	0.769	0.736–0.802
Radiomics nomogram-T2	0.800	0.780–0.820	0.784	0.756–0.812
Radiomics nomogram-MS	0.827	0.809–0.845	0.836	0.815–0.857

### Nomogram Construction and Validation

**Figure 6:**
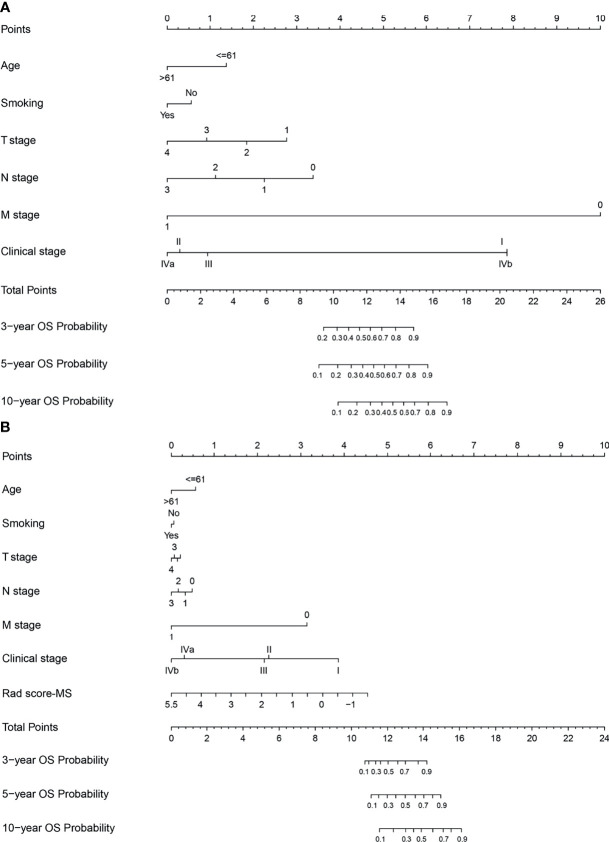
Nomograms developed for the training cohort. **(A)** Clinical nomogram and **(B)** radiomics nomogram-MS.

**Figure 7:**
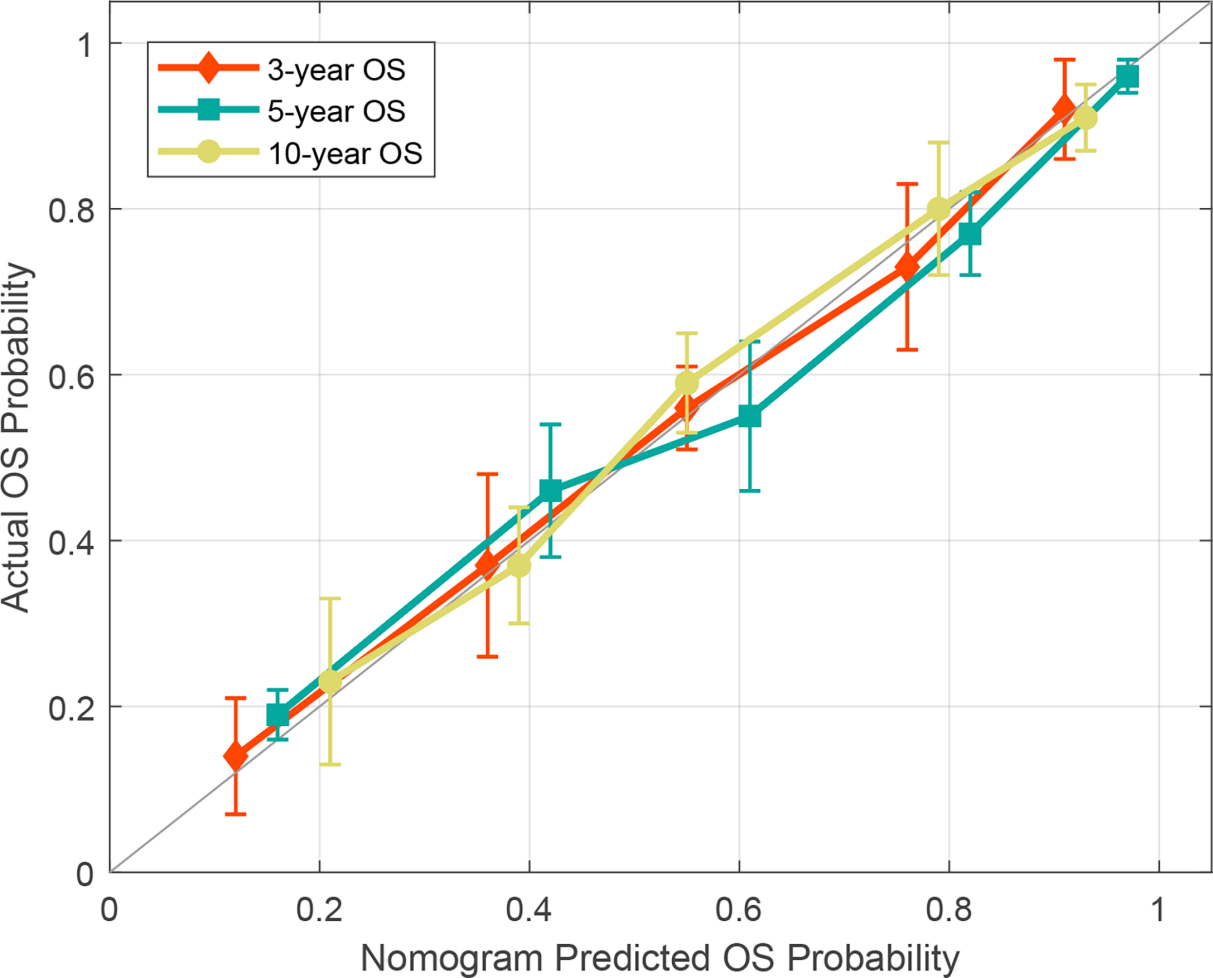
Calibration curve for the radiomics nomogram-MS.

**Figure 8:**
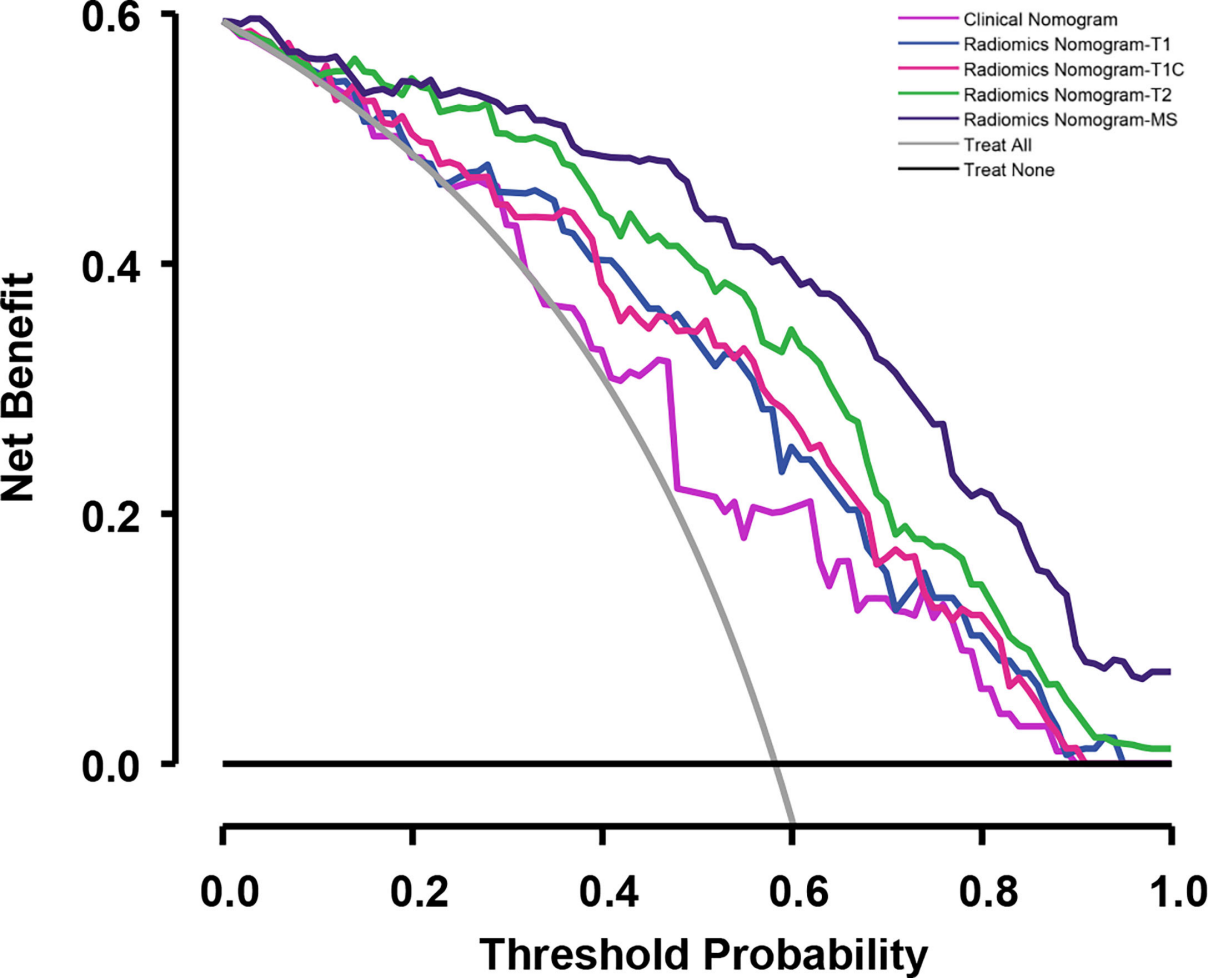
Decision curves for the validation cohort comparing the ability of nomograms to predict overall survival (OS) probability in patients with NPC.

To determine the prediction performance of traditional clinical factors, we first plotted Kaplan–Meier curves for univariate analysis of age, sex, smoking, TNM stage, clinical stage, and pathological type. Significant effects on OS were found for six clinical factors, namely, age, smoking, T stage, N stage, M stage, and clinical stage (all; log-rank test *p* < 0.05). We combined these clinical factors that had a significant effect on OS to construct a clinical nomogram (**Figure 6A**). To determine whether the Rad scores conferred additional benefits for predicting OS, we constructed four radiomics nomograms that incorporated the clinical factors and each of the four Rad scores. These nomograms and the associated Kaplan–Meier curves are presented in the **Supplementary Results**. The C-indices show that radiomics nomogram-MS has optimal discrimination power for predicting the OS probability (**Table 2**). The radiomics nomogram-MS is shown in **Figure 6B**. The radiomics nomogram-MS calibration curves for the 3-, 5-, and 10-year OS probabilities showed good agreement between the predicted and the actual OS probabilities (*p* = 0.146, 0.319, and 0.711, for 3, 5, and 10 years, respectively; **Figure 7**). To determine the clinical utility of the four nomograms, we plotted the decision curves (**Figure 8**). These decision curves also showed that the radiomics nomogram-MS provided a greater net benefit compared with the other three nomograms.

## Discussion

In this study, we developed four Rad scores and four nomograms for risk stratification and to predict the OS probability of patients with NPC. We found that in both the training and validation cohorts, the discrimination and stratification power of Rad score-MS was superior to Rad score-T1, Rad score-T1C, and Rad score-T2 for predicting OS and distinguishing between patients in the low- and high-risk groups. Although all the Rad scores correlated significantly with OS, the discrimination performance measures (C-indices) for Rad score-T1, Rad score-T1C, and Rad score-T2 were significantly lower than those of the clinical nomogram. This is consistent with the findings of a previous study that reported that clinical risk factors, such as TNM staging, have a remarkable ability to determine the prognosis of NPC (21). However, our study found that MS-MRI Rad scores have a better predictive performance than clinical nomograms, possibly because MS-MRI can stereotactically calculate the heterogeneity and decode the phenotype of tumors. We also found that radiomics nomogram-MS, a combination of the Rad score-MS and clinical factors, provided optimal discrimination and had higher clinical utility than other radiomics and clinical nomograms. This suggests that although radiomics nomograms are useful to clinical physicians for early evaluation of long-term outcomes of NPC, MRI radiomics, particularly radiomics nomogram-MS, are even more beneficial for predicting prognosis.

As an optimal model, the radiomics nomogram-MS consisted of 12 radiomics features and clinical factors. These included three features derived from T1 MRI, four features from T1C MRI, and five features from T2 MRI. The features calculated from the original MRI were the maximum 2D diameter and mesh volume. These two features represent the tumor size and have a similar role to the T stage. Besides, six GLCM-based features, of which most are average and probability type features, have the potential to predict survival. It indicates that intratumoral homogeneity may be the reason for differences in survival. In addition, most of the features in the Rad score-MS were wavelets, similar to the findings of others (22, 23), as well as our previous studies (24, 25). This is likely because the multifrequency decomposition of the original MRI image captured more extensive information about the tumor heterogeneity and phenotype to decode long-term survival rules, which clinicians cannot assess with the naked eye. In addition, a ratio of 353 patients to 12 features can avoid overfitting in the Rad score-MS establishing process. Besides, our box plots and Kaplan–Meier curves demonstrated that significant differences existed in both the training and validation cohorts.

Previous studies have demonstrated that incorporating the Rad score and clinical risk factors can significantly improve the prediction performance of many clinical outcomes related to NPC (26–30). For example, multimodality MRI sequences can be used to subdivide non-metastatic NPC patients into four distinct survival subgroups (28). On the other hand, radiomics features extracted from MS differ according to the type of NPC (30), and the Rad score constructed from pretreatment MS-MRI can reliably predict local recurrence in patients with non-metastatic T4 NPC (27). Therefore, a reasonable hypothesis is that MS-MRI can predict OS, and combining MS-MRI with clinical information can improve prediction performance. Our radiomics nomogram-MS results support this hypothesis. This study also demonstrated that multidimensional information, such as radiomics nomogram-MS combined with MS-MRI radiomics features and clinical factors, produced the highest performance in both the training and validation cohorts. Further analysis using the calibration and decision curves also confirmed the utility and effectiveness of the optimal radiomics nomogram-MS.

One of the goals (and strength) of our study was to determine and compare the predictive performance of individual sequences to that of merged features derived from MS-MRI. We found that merged features derived from MS-MRI performed optimally compared with individual sequences. We also found that for MS-MRI, the C-indices calculated from the Rad score generated by T2 MRI images outperformed the C-indices generated from T1 and T1C images in the training cohort. This is likely because compared with the anatomical morphology displayed by T1 and T1C MRI, T2 MRI can distinctly characterize the soft tissue of tumor regions and, therefore, provide more useful information for survival analysis.

A limitation of this study is that it is retrospective and preliminary. As a result, CT images and treatment strategies were unavailable for all enrolled patients. In addition, follow-up information was not completed for progression-free or local recurrence-free survival. Future studies will need to incorporate completed prediction modes for several clinical endpoints. Besides, an external cohort should be collected in the near future to evaluate the robustness and generalizability of the findings.

## Conclusion

Using pretreatment MRI, we have developed and validated a radiomics signature and nomogram that predicts OS in NPC patients. We conclude that radiomics analysis of MRI images can stratify patients into low- and high-risk groups. Furthermore, the optimized radiomics nomogram that incorporates MS-MRI and clinical factors can serve as a useful tool for the early assessment of the long-term prognosis of patients with NPC.

## Data Availability Statement

The original contributions presented in the study are included in the article/**Supplementary Material**. Further inquiries can be directed to the corresponding authors.

## Ethics Statement

Ethics approval of the present study was obtained from the Institutional Review Board of the Affiliated Cancer Hospital of Xinjiang Medical University (No. K-2021022).

## Author Contributions

KL, QQ, YY, and RW contributed to the conception and design of the study. KL organized the database. KL and QQ performed the statistical analysis. KL and QQ wrote the first draft of the manuscript. YQ, TC, DZ, and LH wrote sections of the manuscript. All authors contributed to manuscript revision, read, and approved the submitted version.

## Funding

This work was supported by the Science and Technology Foundation of Xinjiang Uygur Autonomous Region (No. 2020E0265) and the National Natural Science Foundation of China (Nos. 82072094 and 82001902).

## Conflict of Interest

The authors declare that the research was conducted in the absence of any commercial or financial relationships that could be construed as a potential conflict of interest.

## Publisher’s Note

All claims expressed in this article are solely those of the authors and do not necessarily represent those of their affiliated organizations, or those of the publisher, the editors and the reviewers. Any product that may be evaluated in this article, or claim that may be made by its manufacturer, is not guaranteed or endorsed by the publisher.
