# Supplementary material for: Radiomics Nomogram Based on Multiple-Sequence Magnetic Resonance Imaging Predicts Long-Term Survival in Patients Diagnosed With Nasopharyngeal Carcinoma
Source: Front Oncol. 2022 Apr 7;12:852348. doi: 10.3389/fonc.2022.852348 (PMC9021720; doi:10.3389/fonc.2022.852348)
Supplement: Supplementary file 1 [file DataSheet_1.docx]

**Development and validation radiomics nomogram incorporate multiple sequences magnetic resonance imaging for survival prediction of patients with nasopharyngeal carcinoma**

Kai Liu, et al.

**Supplementary Method**

**
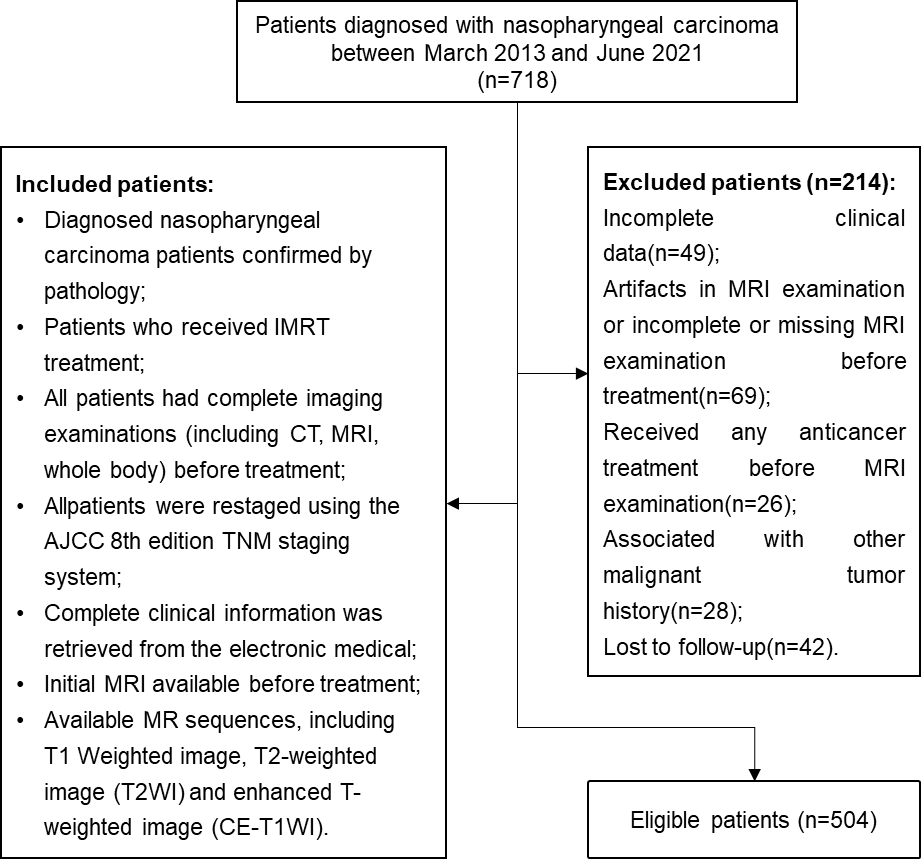
**

**Figure S1.** Flowchart of enrolled nasopharyngeal carcinoma patients in this study.

**Supplementary Results**


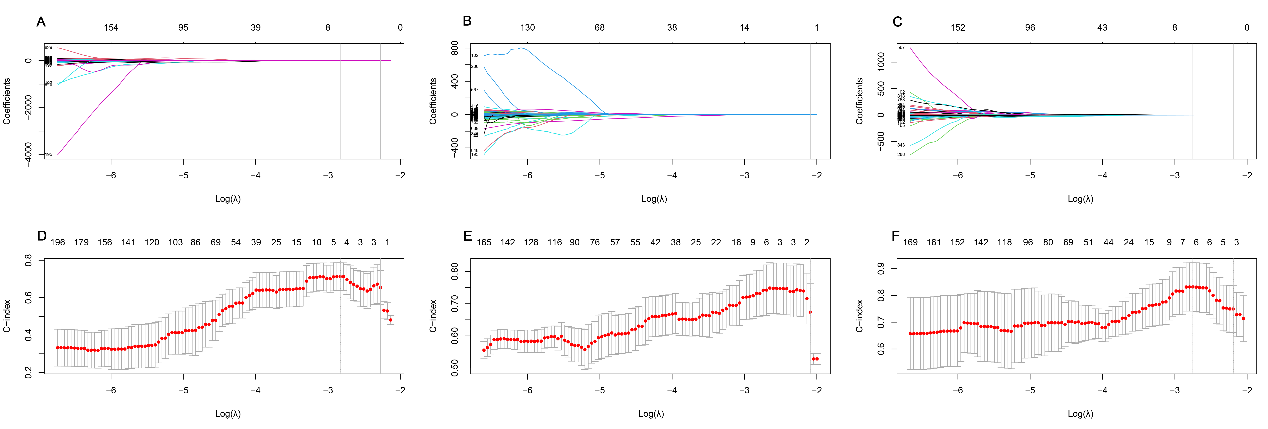


**Figure S2.** Feature selection using the least absolute shrinkage and selection operator (LASSO) with a cox regression model. (A), (B), and (C) The LASSO coefficient profile was plotted using coefficients against log($\lambda$). (D), (E), and (F) Tuning parameter against parameter log($\lambda$). The c-index were depicted with corresponding $\lambda$. Vertical lines are maximum and 1-standard criteria, respectively. (A) and (D), (B) and (E), and (C) and (F) are feature selection results of T1, T1C, and T2, respectively.


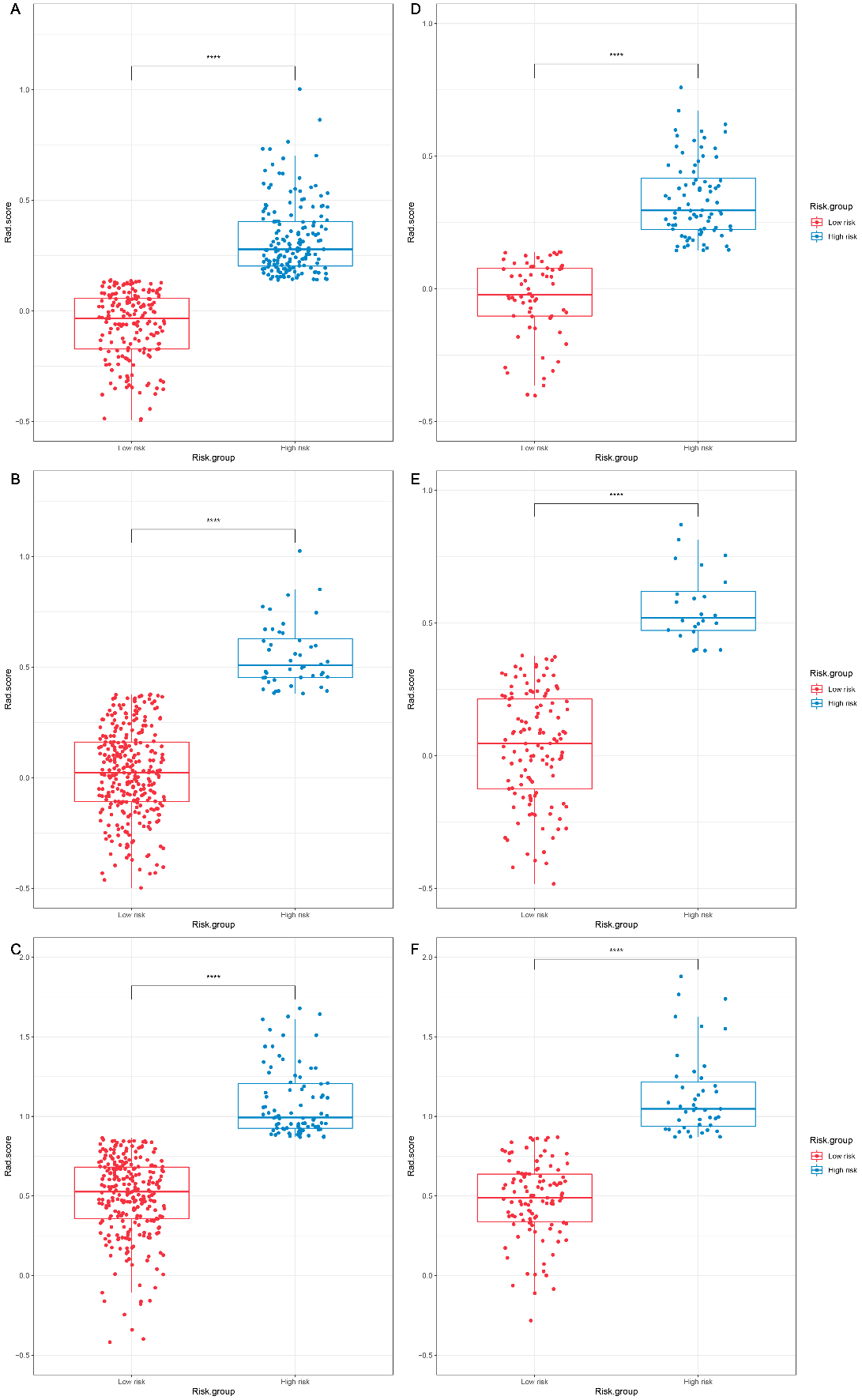


**Figure S3.** Boxplots of risk groups divided by Rad score-MS in (A), (B), and (C) training cohort and (D), (E), and (F) validation cohort. (A) and (D), (B) and (E), and (C) and (F) are boxplots of Rad score-T1, Rad score-T1C, and Rad score-T2, respectively. **** represents p-value<0.0001.


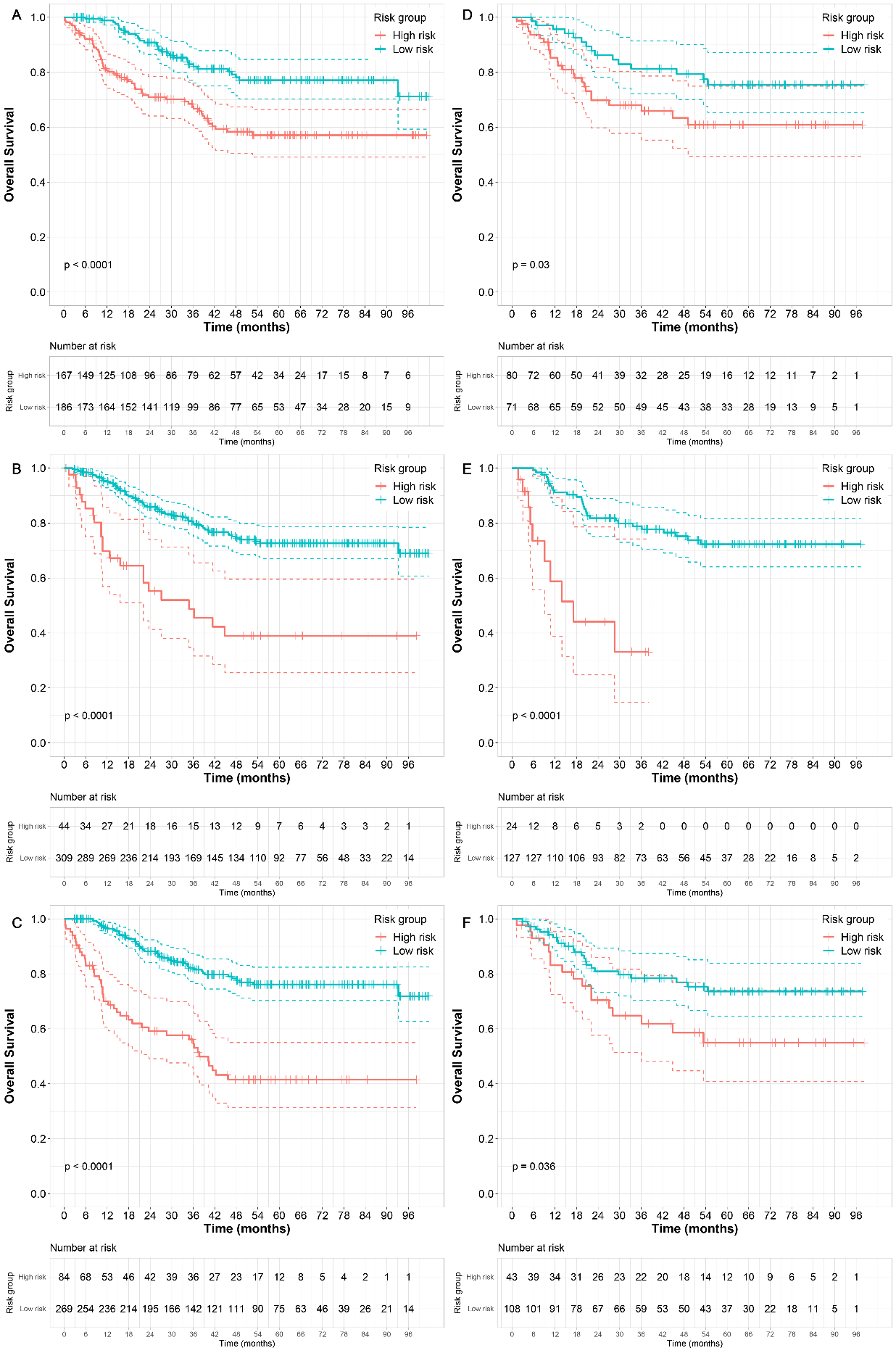


**Figure S4.** Kaplan-Meier survival analysis of high risk and low risk groups of Rad score in (A), (B), and (C) training cohort and (D), (E), and (F) validation cohort. (A) and (D), (B) and (E), and (C) and (F) are Kaplan-Meier cueves of Rad score-T1, Rad score-T1C, and Rad score-T2, respectively. Dashed line in the two-sided CI of survival curves.


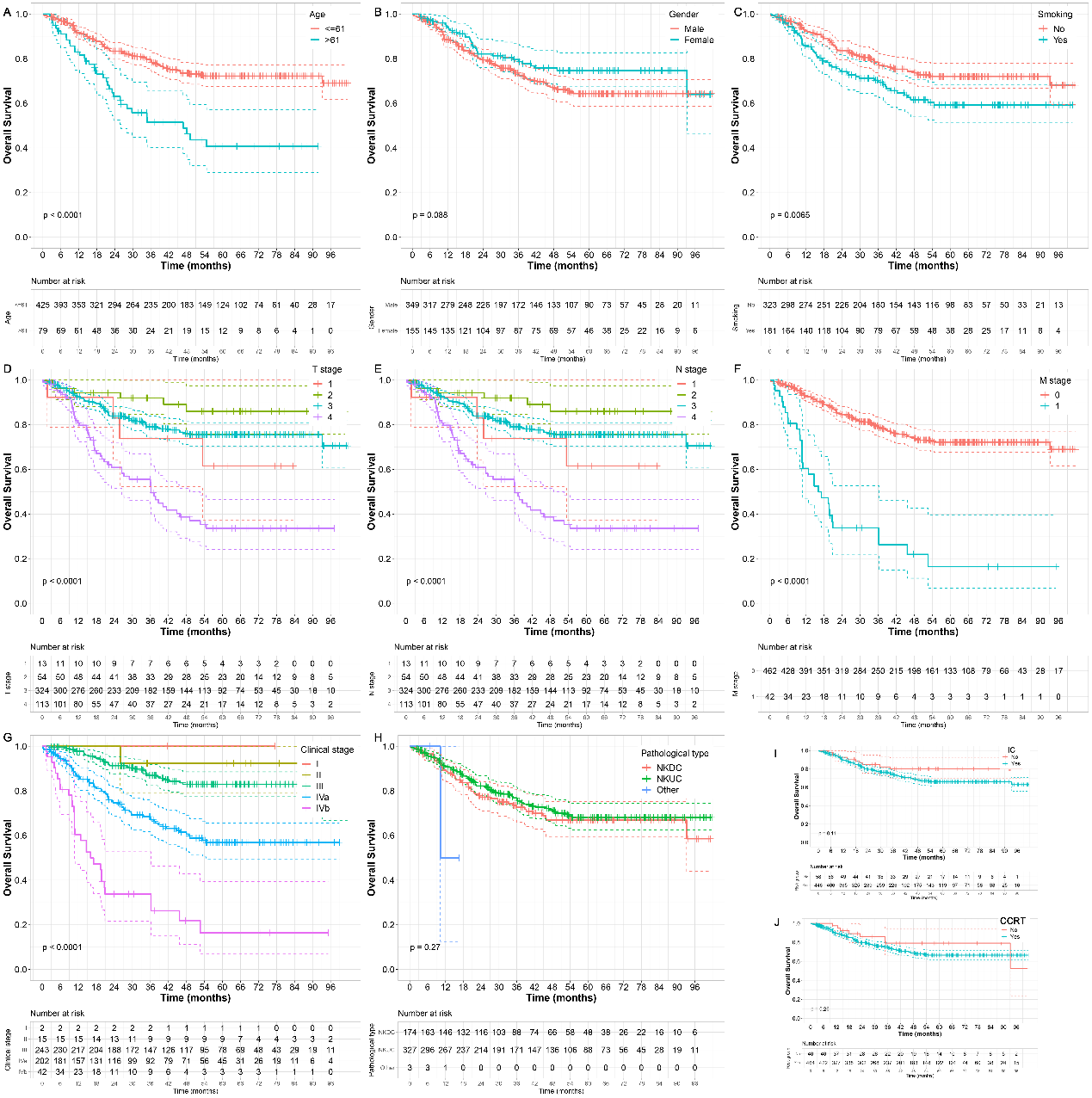


**Figure S5.** Kaplan-Meier curves of clinical factors. The cutoff point of Age was generated using X-tile software.


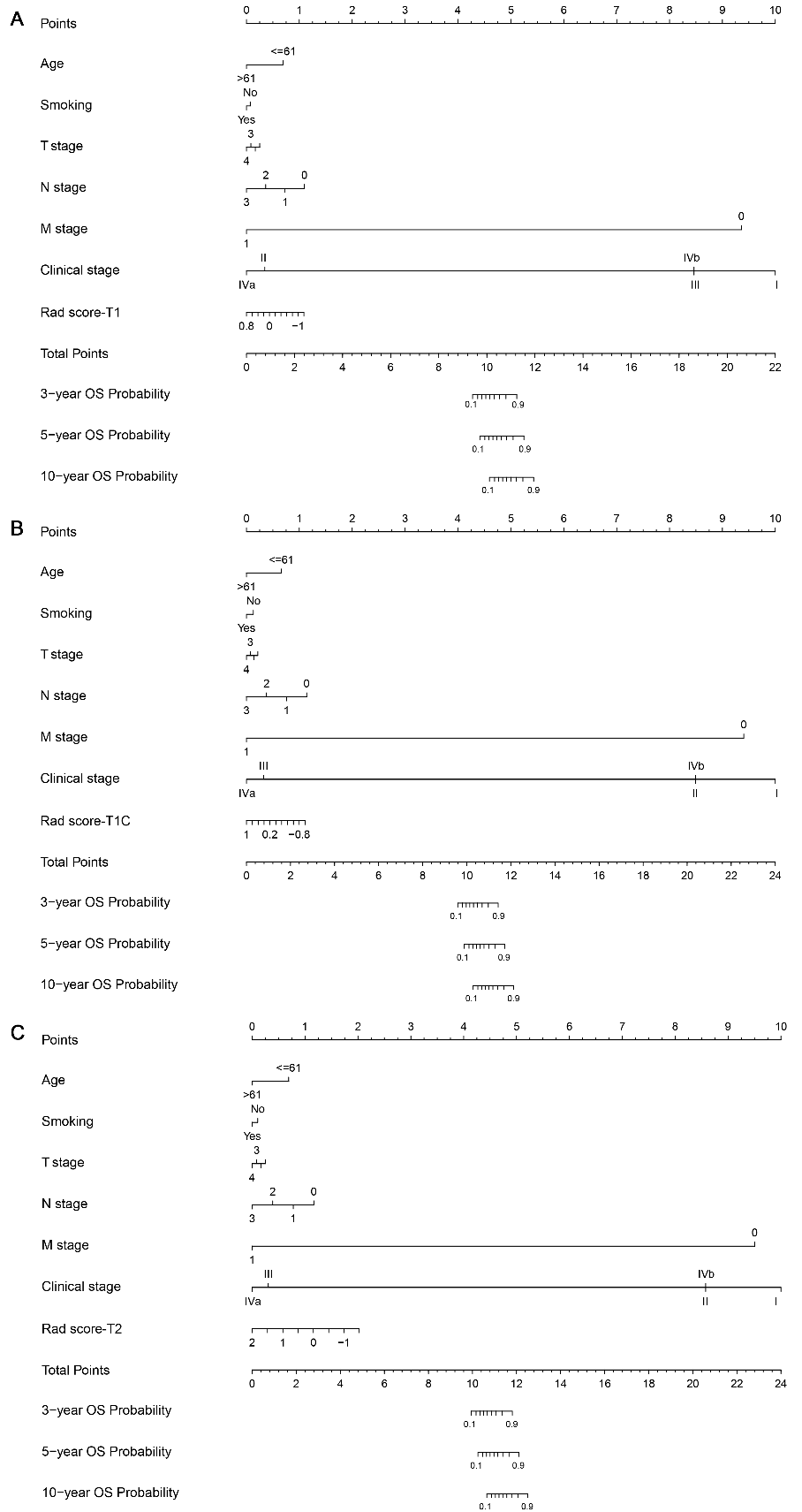


**Figure S6.** Nomograms built in this study, including (A) radiomics nomogram-T1, (B) radiomics nomogram-T1C, and (C) radiomics nomogram-T2.
